# Author Correction: Identifying multiple sclerosis subtypes using unsupervised machine learning and MRI data

**DOI:** 10.1038/s41467-021-23538-6

**Published:** 2021-05-20

**Authors:** Arman Eshaghi, Alexandra L. Young, Peter A. Wijeratne, Ferran Prados, Douglas L. Arnold, Sridar Narayanan, Charles R. G. Guttmann, Frederik Barkhof, Daniel C. Alexander, Alan J. Thompson, Declan Chard, Olga Ciccarelli

**Affiliations:** 1 Queen Square Multiple Sclerosis Centre, Department of Neuroinflammation, UCL Queen Square Institute of Neurology, Faculty of Brain Sciences, University College London, London, UK; 2 Centre for Medical Image Computing (CMIC), Department of Computer Science, Faculty of Engineering Sciences, University College London, London, UK; 3 Department of Neuroimaging, Institute of Psychiatry, Psychology and Neuroscience, King’s College London, London, UK; 4 e-Health Centre, Universitat Oberta de Catalunya, Barcelona, Spain; 5 McConnell Brain Imaging Centre, Montreal Neurological Institute, McGill University, Montreal, QC Canada; 6 Center for Neurological Imaging, Brigham and Women’s Hospital, Harvard Medical School, Boston, MA USA; 7 VU University Medical Centre, Amsterdam, The Netherlands; 8 Department of Brain Repair and Rehabilitation, UCL Queen Square Institute of Neurology, Faculty of Brain Sciences, University College London, London, UK; 9 National Institute for Health Research University College London Hospitals, Biomedical Research Centre, London, UK

**Keywords:** Functional magnetic resonance imaging, Learning algorithms, Multiple sclerosis, Multiple sclerosis

Correction to: *Nature Communications* 10.1038/s41467-021-22265-2, published online 6 April 2021.

The original version of this Article contained an error in Fig. 2, in which the plot shown in panel c was inadvertently duplicated in panel d. The correct version of Fig. 2 is:



which replaces the previous incorrect version:



