# Supplementary material for: Identifying multiple sclerosis subtypes using unsupervised machine learning and MRI data
Source: Nat Commun. 2021 Apr 6;12:2078. doi: 10.1038/s41467-021-22265-2 (PMC8024377; doi:10.1038/s41467-021-22265-2)
Supplement: Supplementary file 1 — Supplementary Information [file 41467_2021_22265_MOESM1_ESM.pdf]

Supplemental material

Supplemental Methods

Brain MRI Protocol

MRI protocols differed between trials and individual trials acquired data under their pre-defined protocols. We included brain 2D or 3D T1-weighted, fluid attenuated inversion recovery (FLAIR), and T2-weighted MRI scans. Table 1 shows the list of included trials with corresponding publications that reported details of MRI protocol.

In the CLIMB study, in which the MRI protocol had changed over time from 2D T1-weighted MRI to 3D, we only included more recent 3D T1 weighted MRI data.

Image analysis

Brain MRI data handling

We checked and labelled the sequence of MRI scans by visually inspecting nine slices of each MRI scan (three axial, three sagittal, and three coronal slices) with equal slice intervals from the coordinates of the "centre of gravity" of each scan. The criteria to define an MRI visit as eligible was the presence of T1-weighted, T2-weighted, and T2-FLAIR MRI modalities with coverage of the brain and the cerebellum. We organised and uploaded MRI data to an XNAT server (version 1.7.4)[1]. We implemented our image analysis pipeline inside XNAT with Nipype version 1.1.4 to enable large-scale high-throughput computing[2].

Initial quality control and inclusion criteria of MRI scans

Quality assurance data was provided by sponsors of each clinical trial, and we excluded MRI data where a quality issue (e.g., acquisition artefact) was flagged. AE also reviewed all scans to ensure coverage of the whole brain, including the

cerebellum, and confirm that there were no additional visible image artefacts that could affect scan processing.

Regional brain volume calculation

We aimed to analyse scans to extract volumes of the grey matter regions according to an established brain atlas developed by Klein and Tourville (Neuromorphometrics, [52], see above for the list)[3]. We chose a cross-sectional, rather than a longitudinal image processing pipeline, to ensure that our subtyping models can be used prospectively in the real-world datasets in which (future) follow up data are not yet acquired. We adapted our established MRI analysis pipeline, which we had previously validated in clinical trials and observational cohorts as explained elsewhere in detail[4,5]. Briefly, it included intensity inhomogeneity correction of the T1-weighted MRI with ITK version 5.0 N4-bias field correction algorithm with Advanced Normalization Tools (ANTs)[6], automatic segmentation of hyperintense lesions of the T2-FLAIR sequence using the consensus (intersection) mask of two different methods (the regression based method in Lesion Segmentation Toolbox version 2.0.15[7] and a deep convolutional neural network based method in DeepMedic version 0.7.1[8] , trained and validated previously with manual lesion masks from MS patients), rigid registration of FLAIR to T1-weighted MRI with co-registration of the FLAIR lesion masks to T1-weighted MRI using ANTs version 2.1.0, and lesion filling with NiftySeg version 1.0[9]. We segmented and parcellated the brain into Neuromorphometrics atlas regions on lesion-filled T1-weighted scans using the Geodesic Information Flows (GIF) software version 3.0[10]. We used a modified version of this pipeline for the Siena cohort, ARPEGGIO and lamotrigine trials which did not have FLAIR but whose investigators had provided manually delineated lesion masks.

T1/T2 ratio calculation of the normal-appearing white matter regions

Lesion masks or brain volumes do not provide any quantitative information on microstructural changes in the white matter. We therefore chose T1/T2 ratio as a measure of extra-lesional white matter changes, because T1 and T2-weighted MRI are widely available in clinical trials and clinical practice (as opposed to more advanced MRI sequences such as diffusion imaging or magnetisation transfer ratio). T1/T2 ratio is an extensively used measures of microstructural changes[11,12]. We adapted available pipelines from the Human Connectome Project to calculate T1/T2 ratio maps for all trials[13] and Ganzetti and colleagues method in T1/T2 calculation[14]. We corrected for intensity inhomogeneity in T1 and T2-weighted MRI scans with N4 bias field correction algorithm. Next, we rigid-registered T1 and T2-weighted scans in a symmetric space, such that both modalities equally underwent only one interpolation to minimise interpolation artefacts. We normalised the intensity of each modality separately as explained in Ganzetti et al. Ganzetti et al. used measurements of the vitreous humour and temporal muscles to normalise T1/T2 ratios; however, because of data anonymisation, subjects' eyes were removed from the scans in several of the clinical trials included in this study. Therefore, we used T1/T2 ratio of the ventricular CSF to normalise individual T1/T2 ratios. When we compared ventricular CSF T1/T2 ratios between MS patients and controls, no significant differences were detected. We calculated the T1/T2 ratio and normalised its value against the average T1/T2 ratio in the ventricles with the co-registered ventricular masks obtained from the segmentation maps calculated from lesion-filled T1 scans (explained above). We extracted T1/T2 ratio from bilateral normal-appearing white matter regions (see above for list of regions) after we removed co-registered lesions segmented in FLAIR from the white

matter regions, which we refer to as normal-appearing T1/T2 ratio throughout this manuscript. Since the T1/T2 ratio in the grey matter regions were highly correlated with grey matter volumetric results, we did not include any T1/T2 ratio in the grey matter in our models.

Quality control

We developed a pipeline to check the quality of results of our pipeline by automatically generating 18 images from segmentation results, lesion segmentations, and registration results which we manually reviewed. We re-ran image analysis pipeline where we identified mis-registrations or faulty segmentations. We did not exclude any visit in clinical trials to perform an intention-to-treat analysis in individuals who met the minimal MRI criteria (which was availability of T1-, T2-weighted, and FLAIR).

Supplemental Statistical Analysis

Centre effects and 2D or 3D data acquisition effects

From the 19 data sets in the training and validation sets, 15 were multi-centre, which means that their MRI data were acquired by two or more scanners. Eight data sets were acquired by a 3D and the remaining 11 with a 2D T1-weighted acquisition protocol. To compare “centre” effects with “subtype” effects, using all data from training and validation datasets, we fitted hierarchical mixed effects models in which MRI variables were outcome, “centre” and “subtype” were predictors, and “study” was the random effects variable. We also determined whether 2D versus 3D MRI protocols, or SuStaIn model subtypes explain the majority of variation in regional brain tissue volumes. To compare the effects of 3D vs 2D MRI data acquisition, we used a mixed effects model, in which data acquisition (2D or 3D) and MRI-derived subtypes were predictors and regional brain volumes were outcomes. We quantified the effect size for each variable and compared them using general linear model contrasts with the multcomp package in R.

Comparison of MRI subtypes and established imaging measures in prediction of CDP

We assessed whether the three MRI derived subtypes provide value to the known imaging outcomes of lesion load and whole brain volume. We divided patients based on these two variables in three equal groups and compared the hazard ratios with the MRI-derived subtypes in a Cox regression model. In this model we also included baseline EDSS, disease duration, and age as confounders.

Reliability and stability of SuStaIn models: longitudinal subtyping

In addition to subtyping patients at baseline, we trained our model on the baseline subjects and predicted the probability of subtype membership for the available patient visits over time (34,172 visits). We reported the number of subjects who preserved the subtype membership. To calculate the annual rate of change in SuStaIn stages for each data-driven subtype, we fitted a mixed-effects model in which the SuStaIn stage was an outcome variable and time was the independent variable (fixed effects). In these models to adjust for hierarchical repeated measures, we defined nested random effects in which 'time' variable was nested in the 'subject' variable. To calculate longitudinal cortical atrophy in each subtype we used a similar mixed effects model and log-transformed the cortical volumes to obtain the annual percentage volume change.

Supplemental Results

Demographic characteristics of the training and validation datasets

When we compared the training data set with the validation data set, patients in the validation data set were younger (average difference of 3.1 years, $p<0.001$), had shorter disease duration (average difference of 6 months, $p=0.001$), and were less disabled (0.5 difference in Expanded Disability Status Scale (EDSS), $p<0.001$) than those in the training dataset.

MRI Processing: calculating normal ageing and gender effects using datasets of healthy volunteers

We obtained 18 MRI variables which were volumes of grey matter lobes and deep grey matter, white matter lesion load, and normal-appearing WM T1/T2 for all patients and healthy volunteers. To estimate and adjust for demographic variables and ageing effects in MRI, we used two data sets from 14,928 healthy volunteers which covered a wide range of age (23.5 to 70 years, 13,823 from the UK Biobank and 1,105 from the Human Connectome Project; 7,965 women and 6,963 men). The mean age was 28.9 years (standard deviation=3.62) for the Human Connectome Project and 54.9 years (standard deviation=7.49) for the UK Biobank. We used healthy controls' datasets to calculate linear and non-linear effects of age, gender, and total intracranial volume and adjusted MRI variables in patients for these effects. Of the 18 adjusted MRI variables, 13 were associated with a moderate to large effect size when patients at baseline visits were compared with healthy controls, and, therefore, were selected and entered into SuStaIn (Supplementary Figure 1**)**. Selected variables were volumes of the occipital, parietal, temporal, limbic and frontal grey matter, and deep grey matter;

total white matter lesion volume; T1/T2 ratio in the corpus callosum, frontal, temporal, parietal, cingulate bundle and cerebellar normal-appearing white matter (NAWM) regions.

Defining the optimal number of subtypes: model selection

We fitted models that had one to five subtypes for 14 cross-validation folds (total of 70 models, Supplementary Figure 3 and Supplementary Figure 4). We used Cross Validation Information Criteria (CVIC) to choose the most optimal number. The three-subtype model had the optimal average CVIC (minimum value across models up to five subtypes) across tested models.

“Dataset” and “centre” effects

We found that the subtypes were highly consistent across datasets in the training data set, despite different RCTs and trial protocols. In particular, the average measure of agreement (or Bhattacharyya coefficient) of the posterior distribution of the estimated sequences for each subtype across all cross-validation folds were as follows: 0.94 (standard deviation±0.03) for the cortex-led subtype, 0.94 (standard deviation ±0.02) for the NAWM-led subtype, and 0.96 (standard deviation ±0.02) for the lesion-led subtype, suggesting excellent agreement across trials. When we looked at the effects of centre inside each dataset on MRI-derived subtypes, the EDSS and MRI measures were significantly more strongly associated to “subtype” effect than the “centre effect” (see Supplemental Results).

Centre vs subtype effects: Subtype was more strongly associated with clinical and imaging outcomes than the centre

MRI and clinical data in training datasets were acquired at 772 different centres. EDSS was more strongly associated with subtype than centre (difference in standardised β= 0.04, standard error = 0.009, p<0.001). Similarly, when looking at the 13 MRI measures, their standardised β coefficients were significantly larger than centre coefficients, which means that they were more strongly associated with subtype than centre (all p values < 0.001). When we compared the effects of 2D or 3D acquisition of T1-weighted MRI vs MRI-derived subtypes on brain volumes, the magnitude of effect sizes was, 1.5 times (for the parietal lobe) and 9.9 times (for the deep grey matter) larger for MRI-derived subtypes than 2D or 3D MRI acquisitions.

Relationship of subtype classification certainty to 24-week CDP

In the placebo arms of the validation data set, there were significant correlations across classification certainties and time to 24-week CDP: A higher certainty of lesion-led and NAWM-led classification were correlated with a longer time to CDP (Pearson's r = 0.15, p=0.02 for lesion led and r=0.23, p=0.01 for NAWM-led). There was no correlation between the certainty of cortex-led classification and CDP (p=0.96).

Comparison across MRI subtypes and established imaging measures in predicting CDP

The MRI-derived subtypes were significantly associated with the time to 24-week CDP while the lesion volumes or brain volumes were not (in the same model): MRI-derived subtypes (hazard ratio (HR)=1.15, SE=0.06, p=0.04), total brain volume (HR=1.13, SE=0.07, p=0.1), and lesion load (HR=1.08, SE=0.07, p=0.25).

Consistency of the subtype membership over time

Presented results so far have been on subtypes detected using the baseline MRI scans. We looked at longitudinal stability of subtype membership, too, to examine the reliability of SuStaIn. We divided the participants into high and low certainty groups using the median classification certainty as the cut-off, which was 96.3%, at baseline. In the high certainty group, 11.4% of participants (542 out of 4740 patients) changed subtype during the study. In the low certainty group, 24% of participants (1169 out of 4739) changed subtype.

Supplementary references

1. Marcus, D. S., Olsen, T. R., Ramaratnam, M. & Buckner, R. L. The Extensible Neuroimaging Archive Toolkit: an informatics platform for managing, exploring, and sharing neuroimaging data. *Neuroinformatics* **5**, 11–34 (2007).
2. Gorgolewski, K. *et al.* Nipype: a flexible, lightweight and extensible neuroimaging data processing framework in python. *Front Neuroinform* **5**, 13 (2011).
3. Klein, A. & Tourville, J. 101 labeled brain images and a consistent human cortical labeling protocol. *Frontiers in Neuroscience* **6**, (2012).
4. Eshaghi, A. *et al.* Deep gray matter volume loss drives disability worsening in multiple sclerosis. *Ann. Neurol.* **83**, 210–222 (2018).
5. Eshaghi, A. *et al.* Applying causal models to explore the mechanism of action of simvastatin in progressive multiple sclerosis. *Proc. Natl. Acad. Sci. U.S.A.* **116**, 11020–11027 (2019).
6. Tustison, N. J. *et al.* N4ITK: improved N3 bias correction. *IEEE Trans Med Imaging* **29**, 1310–1320 (2010).
7. Schmidt, P. *et al.* An automated tool for detection of FLAIR-hyperintense white-matter lesions in Multiple Sclerosis. *Neuroimage* **59**, 3774–3783 (2012).
8. Kamnitsas, K. *et al.* Efficient multi-scale 3D CNN with fully connected CRF for accurate brain lesion segmentation. *Medical Image Analysis* **36**, 61–78 (2017).
9. Prados, F. *et al.* A multi-time-point modality-agnostic patch-based method for lesion filling in multiple sclerosis. *Neuroimage* **139**, 376–384 (2016).
10. Cardoso, M. J. *et al.* Geodesic information flows: spatially-variant graphs and their application to segmentation and fusion. *IEEE Trans Med Imaging* **34**, 1976–1988 (2015).

11. Righart, R. *et al.* Cortical pathology in multiple sclerosis detected by the T1/T2-weighted ratio from routine magnetic resonance imaging. *Ann. Neurol.* (2017) doi:10.1002/ana.25020.
12. Glasser, M. F. *et al.* A multi-modal parcellation of human cerebral cortex. *Nature* **536**, 171–178 (2016).
13. Glasser, M. F. & Van Essen, D. C. Mapping human cortical areas in vivo based on myelin content as revealed by T1- and T2-weighted MRI. *J. Neurosci.* **31**, 11597–11616 (2011).
14. Ganzetti, M., Wenderoth, N. & Mantini, D. Whole brain myelin mapping using T1- and T2-weighted MR imaging data. *Front. Hum. Neurosci.* **8**, (2014).

International Progressive MS Alliance (PMSA) Investigators of the network

Name	Institution
Douglas L Arnold	McConnell Brain Imaging Centre, Montreal Neurological Institute, McGill University, Montreal, Quebec, Canada
Sridar Narayanan	McConnell Brain Imaging Centre, Montreal Neurological Institute, McGill University, Montreal, Quebec, Canada
Frederik Barkhof	Queen Square Multiple Sclerosis Centre, Department of Neuroinflammation, UCL Queen Square Institute of Neurology, Faculty of Brain Sciences, University College London, WC1B5EH, UK
Olga Ciccarelli	Queen Square Multiple Sclerosis Centre, Department of Neuroinflammation, UCL Queen Square Institute of Neurology, Faculty of Brain Sciences, University College London, WC1B5EH, UK
Declan Chard	Queen Square Multiple Sclerosis Centre, Department of Neuroinflammation, UCL Queen Square Institute of Neurology, Faculty of Brain

	Sciences, University College London, WC1B5EH, UK
Louis Collins	McConnell Brain Imaging Centre, Montreal Neurological Institute, McGill University, Montreal, Quebec, Canada
Tal Arbel	McConnell Brain Imaging Centre, Montreal Neurological Institute, McGill University, Montreal, Quebec, Canada
Charles R.G Guttman	Center for Neurological Imaging, Brigham and Women's Hospital, Harvard Medical School, Massachusetts, USA
Jerry S Wolinsky	McGovern Medical School, The University of Texas Health Science Center at Houston (UTHealth), Houston, Texas, USA
Garry R Cutter	University of Alabama at Birmingham School of Public Health, USA
Nicola De Stefano	University of Siena, Italy
Maria Pia Sormani	University of Genoa, Italy
Ludwig Kappos	University Hospital Basel, Switzerland
Jack H Simon	Oregon Health and Sciences University, Portland Veterans Affairs Medical Center, Oregon, USA
Jeremy Chataway	Queen Square Multiple Sclerosis Centre, Department of Neuroinflammation, UCL Queen Square Institute of Neurology, Faculty of Brain Sciences, University College London, WC1B5EH, UK
Raj Kapoor	Queen Square Multiple Sclerosis Centre, Department of Neuroinflammation, UCL Queen Square Institute of Neurology, Faculty of Brain

	Sciences, University College London, WC1B5EH, UK
Howard L. Weiner (CLIMB Investigator)	Brigham and Women's Hospital, Ann Romney Center for Neurologic Diseases, Department of Neurology, Boston, MA, 02115
Tanuja Chitnis (CLIMB Investigator)	Brigham and Women's Hospital, Ann Romney Center for Neurologic Diseases, Department of Neurology, Boston, MA, 02115
Rohit Bakshi (CLIMB Investigator)	Brigham and Women's Hospital, Ann Romney Center for Neurologic Diseases, Department of Neurology, Boston, MA, 02115

MS-SMART Investigators

Jeremy Chataway, Claudia A M Gandini Wheeler-Kingshott, Floriana De Angelis, Domenico Plantone, Anisha Doshi, Nevin John, Thomas Williams, Jonathan Stutters, Ferran Prados Carrasco, David MacManus, Frederik Barkhof, Sebastien Ourselin, Marie Braisher, Tiggy Beyene, Vanessa Bassan, Alvin Zapata (Queen Square Multiple Sclerosis Centre, University College London and University College London Hospitals NHS Foundation Trust, London, UK); Siddharthan Chandran, Peter Connick, Dawn Lyle, James Cameron, Daisy Mollison, Shuna Colville, Baljean Dhillon (Anne Rowling Regenerative Neurology Clinic, The University of Edinburgh, Royal Infirmary of Edinburgh, NHS Lothian, Edinburgh, UK); Christopher J Weir, Richard A Parker, Moira Ross, Gina Cranswick, Allan Walker, Lorraine Smith (Edinburgh Clinical Trials Unit [ECTU], Usher Institute, University of Edinburgh, Edinburgh, UK); Gavin Giovannoni, Sharmilee Gnanapavan (Blizard Institute, Barts and The London School of Medicine and Dentistry, Queen Mary University, Barts Health NHS Trust, London, UK); Richard Nicholas (Imperial College Healthcare NHS Trust, London, UK); Waqar Rashid, Julia Aram (Brighton and Sussex University Hospitals NHS Trust, Brighton, UK); Helen Ford (Leeds General Infirmary, Leeds Teaching Hospitals NHS Trust, Leeds, UK); Sue H Pavitt (Dental Translational and Clinical Research Unit, University of Leeds, Leeds, UK); James Overell (The Queen Elizabeth University Hospital Glasgow, NHS Greater Glasgow and Clyde, Glasgow, UK); Carolyn Young, Heinke Arndt (The Walton Centre NHS Foundation Trust, Liverpool, UK); Martin Duddy, Joe Guadagno (Royal Victoria Infirmary, The Newcastle upon Tyne Hospital NHS Foundation Trust, Newcastle, UK); Nikolaos Evangelou (Queens Medical Centre, Nottingham University Hospital NHS Trust, Nottingham, UK); Matthew Craner, Jacqueline Palace (John Radcliffe Hospital, Oxford University Hospitals NHS Foundation Trust, Oxford, UK); Jeremy Hobart (Derriford Hospital, University Hospitals Plymouth NHS Trust, Plymouth, UK); Basil Sharrack, David Paling (Royal Hallamshire Hospital, Sheffield Teaching Hospitals NHS Foundation Trust, Sheffield, UK); Clive Hawkins, Seema Kalra (Royal Stoke University Hospital, University Hospitals of North Midlands NHS Trust, Stoke-on-Trent, UK); Brendan McLean (Royal Cornwall Hospitals NHS Trust, Truro, UK); Nigel Stallard (Statistics and Epidemiology, Division of Health Sciences, Warwick Medical School, University of Warwick, Coventry, UK); and Roger Bastow (patient representative).

Supplementary Figures

Supplementary Figure 1. Variable selection.

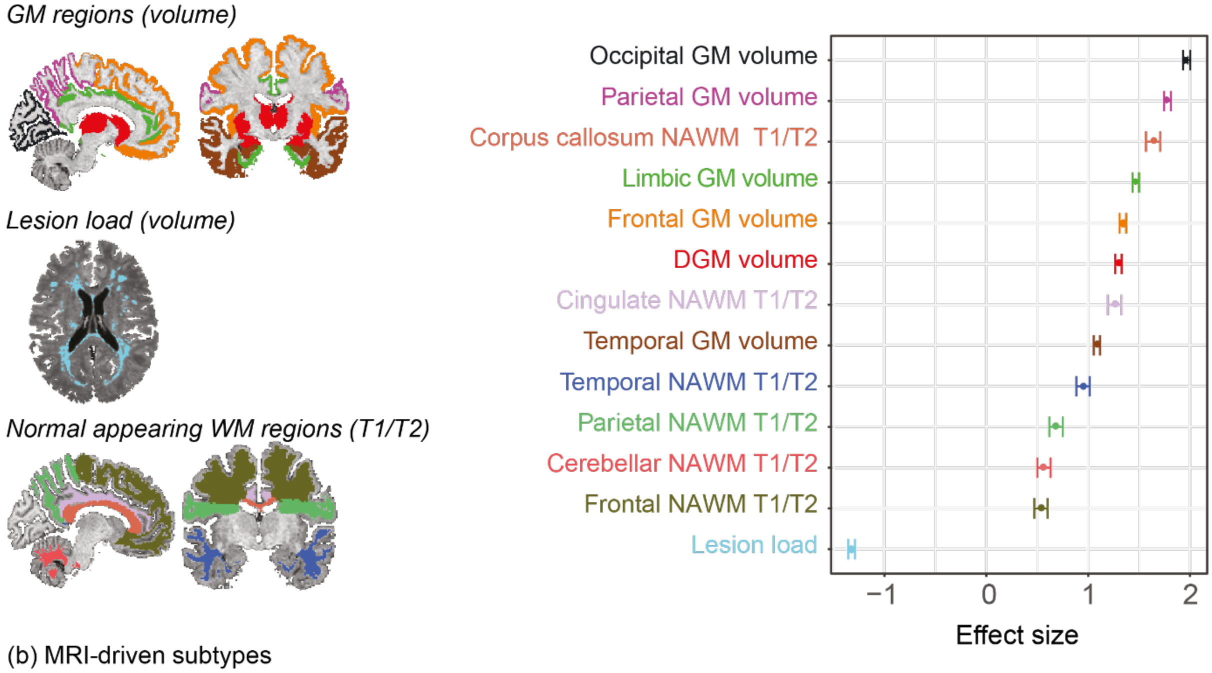

(b) MRI-driven subtypes

(a) We chose variables whose effect size was medium to large (Cohen's d effect size greater than 0.5) when comparing all patients of the training data set (n=6,322) with healthy volunteers (n=14,928). We have overlayed selected 13 variables on a T1-weighted MRI scan of a randomly chosen patient. We used the same colour coding to show selected variables on the brain MRI scan and the right plot. On the right plot, dots (centre measure) represent point estimates of the effect size and error bars represent the 95% confidence interval of the effect size.

Supplementary Figure 2. Positional variance diagram of three data-driven subtypes of multiple sclerosis.

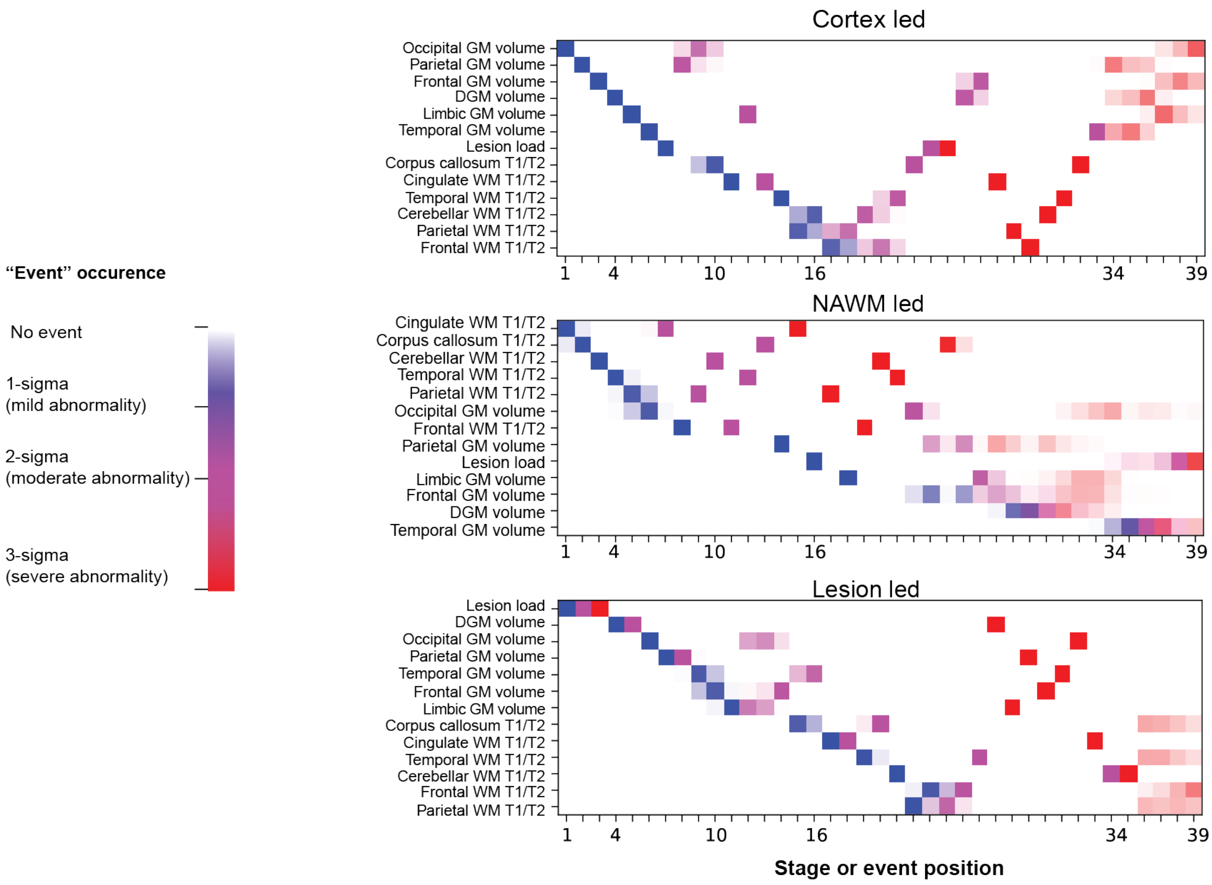

The evolution of variables in each subtype is defined by a continuous z-score model, in which each variable follows a piecewise trajectory over a temporal order (or stage). Positional variance diagram for the three MRI-derived imaging subtypes. The three different colours represent the degree of abnormality based on Z-score (sigma or standard deviation) models: mild=blue, moderate=violet, and severe=red. The colour shades represent the uncertainty associated with each event position in the posterior distribution of 100,000 Markov Chain Monte Carlo samples. A transition from a stage to the next one is different between subtypes; for example a change from stage 4 to 5 refers to the development of mild atrophy in the limbic cortex in the cortex-led subtype, the development of mild abnormality in the parietal white matter T1/T2 ratio and mild

atrophy in the occipital cortex in the NAWM-led subtype, and appearance of atrophy in the deep grey matter in the lesion-led subtype.

Acronyms: DGM, deep grey matter; T1/T2, T1-T2 ratio; WM, white matter; GM, grey matter; DGM, deep grey matter; NAWM, normal-appearing white matter.

Supplementary Figure 3. Model development.

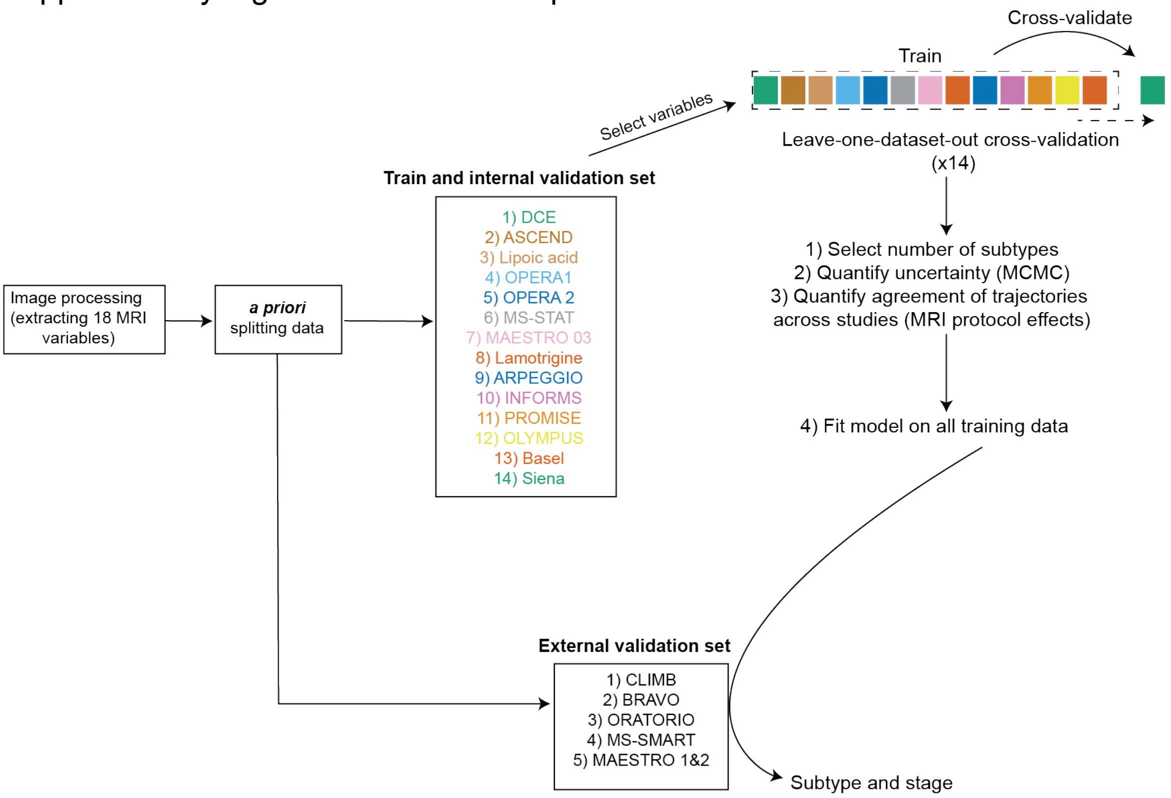

"Raw" MRI data from different data sets underwent a unique image processing pipeline to extract variables (or features) of lobar grey matter volume, visible white matter lesion from FLAIR, and T1/T2 ratio. We used healthy volunteers (UK Biobank and the Human Connectome Project) to adjust MRI measures nuisance variables (see Methods and Supplemental Results), calculate Z-scores, and select MRI variables. *A priori* we split our patient datasets into two separate datasets: 14 datasets in the training dataset, and five datasets for validation: CLIMB (an observational study), BRAVO (a phase 3 RRMS trial), ORATORIO (a phase 3 PPMS trial), MS-SMART (a phase 2 SPMS trial), and MAESTRO 1&2 (a phase 3 SPMS trial).

Abbreviations: MCMC, Markov Chain Monte Carlo; RRMS, relapsing remitting multiple sclerosis; PPMS, primary progressive multiple sclerosis; MRI, magnetic resonance imaging. DCE, DEFINE/CONFIRM/ENDORSE.

Supplementary Figure 4. Leave-one-dataset-out cross-validation and model selection.

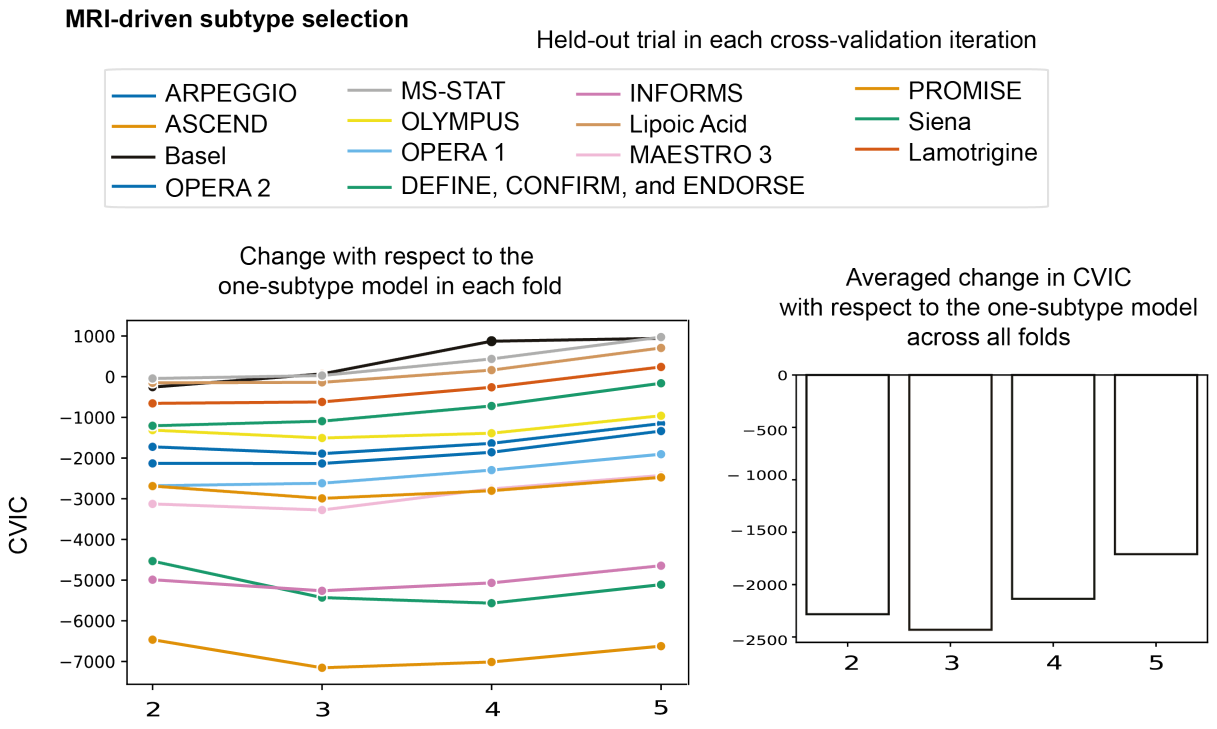

We used leave-one-dataset-out in the training data set of 14 studies, each time leaving one study out and fitting SuStaIn algorithm on the remiaing 13 datasets. We chose the best number of subtypes according to the cross-validation information critiera (CVIC) calcualted from the *left-out* dataset each time (x14). The vertical axis shows the change in CVIC with respect to the one-subtype model. The relative average difference between three-subytpe model and two-subtype model was 147. A relative difference of 6 is considered strong evidence that a model is better than another (Young et al, 2018).
