# Supplementary material for: Identifying multiple sclerosis subtypes using unsupervised machine learning and MRI data
Source: Nat Commun. 2021 Apr 6;12:2078. doi: 10.1038/s41467-021-22265-2 (PMC8024377; doi:10.1038/s41467-021-22265-2)
Supplement: Supplementary file 3 — Reporting Summary [file 41467_2021_22265_MOESM3_ESM.pdf]

nature research

Corresponding author(s): Arman Eshaghi

Last updated by author(s): 25 Feb 2021

Reporting Summary

Nature Research wishes to improve the reproducibility of the work that we publish. This form provides structure for consistency and transparency in reporting. For further information on Nature Research policies, see our Editorial Policies and the Editorial Policy Checklist.

Statistics

For all statistical analyses, confirm that the following items are present in the figure legend, table legend, main text, or Methods section.

n/a	Confirmed	
☐	☒	The exact sample size (*n*) for each experimental group/condition, given as a discrete number and unit of measurement
☐	☒	A statement on whether measurements were taken from distinct samples or whether the same sample was measured repeatedly
☐	☒	The statistical test(s) used AND whether they are one- or two-sided<br>*Only common tests should be described solely by name; describe more complex techniques in the Methods section.*
☐	☒	A description of all covariates tested
☐	☒	A description of any assumptions or corrections, such as tests of normality and adjustment for multiple comparisons
☐	☒	A full description of the statistical parameters including central tendency (e.g. means) or other basic estimates (e.g. regression coefficient) AND variation (e.g. standard deviation) or associated estimates of uncertainty (e.g. confidence intervals)
☐	☒	For null hypothesis testing, the test statistic (e.g. *F*, *t*, *r*) with confidence intervals, effect sizes, degrees of freedom and *P* value noted<br>*Give P values as exact values whenever suitable.*
☐	☒	For Bayesian analysis, information on the choice of priors and Markov chain Monte Carlo settings
☐	☒	For hierarchical and complex designs, identification of the appropriate level for tests and full reporting of outcomes
☐	☒	Estimates of effect sizes (e.g. Cohen's *d*, Pearson's *r*), indicating how they were calculated

Our web collection on statistics for biologists contains articles on many of the points above.

Software and code

Policy information about availability of computer code

Data collection	Not applicable. This manuscript has re-used pre-existing data so no software was used in data collection by authors.
Data analysis	A Code Availability section was added to the manuscript. The link to the code is: https://github.com/ucl-pond/pySuStaIn.<br><br>DeepMedic version 0.7.1 was used (https://github.com/deepmedic/deepmedic).<br><br>NiftySeg version 1.0 and GIF version 3.0 were used. (https://github.com/KCL-BMEIS/NiftySeg)<br><br>Advanced Normalization Tools (ANTs) version 2.3.0: https://github.com/ANTsX/ANTs<br><br>FSL version 6.0 : https://fsl.fmrib.ox.ac.uk/fsl/fslwiki<br><br>We used NLME package version 3.1 and Survival package version 2.44 inside R version 3.6.0 for statistical analysis (all are available at https://cran.r-project.org/).

For manuscripts utilizing custom algorithms or software that are central to the research but not yet described in published literature, software must be made available to editors and reviewers. We strongly encourage code deposition in a community repository (e.g. GitHub). See the Nature Research guidelines for submitting code & software for further information.

Data

Policy information about availability of data

All manuscripts must include a data availability statement. This statement should provide the following information, where applicable:

- Accession codes, unique identifiers, or web links for publicly available datasets
- A list of figures that have associated raw data
- A description of any restrictions on data availability

Unprocessed clinical trial data are not publicly available, because controlled by commercial stakeholders (listed in references in the Table 1) and request to access data should be forwarded to data controllers. Processed data can be requested by qualified investigators from the corresponding author. Data from UK Biobank and Human Connectome Project (see below) are publicly available. Links to the publicly available data are given under Data Availability Statement.

Field-specific reporting

Please select the one below that is the best fit for your research. If you are not sure, read the appropriate sections before making your selection.

☒ Life sciences ☐ Behavioural & social sciences ☐ Ecological, evolutionary & environmental sciences

For a reference copy of the document with all sections, see nature.com/documents/nr-reporting-summary-flat.pdf

Life sciences study design

All studies must disclose on these points even when the disclosure is negative.

Sample size	We retrospectively included all the major clinical trials in multiple sclerosis whose sponsors agreed to provide raw clinical and imaging data from all individuals in their studies.
Data exclusions	We did not exclude any scans and performed intention-to-treat analysis. We filtered the datasets to include those visits that had a minimal MRI criteria (see Supplemental Material).
Replication	We have used cross-validation and external testing on more than 9,390 patients to ensure replication. All attempts to replicate findings were successful, which means that data driven subtypes were detected in new data sets with similar patterns.
Randomization	All the clinical trials included are double-blind randomised controlled trials. There are three observational cohorts, that are not randomised. We replicated findings of the observational cohorts in RCTS to ensure that they findings were not confounded by well-known limitations in such cohorts. We did not use observational cohorts for treatment response calculation to avoid any bias in this regard.
Blinding	Given the retrospective and analytical nature of this study, the lead scientist and data analyst (Arman Eshaghi) was blinded to treatment allocation during the image processing

Reporting for specific materials, systems and methods

We require information from authors about some types of materials, experimental systems and methods used in many studies. Here, indicate whether each material, system or method listed is relevant to your study. If you are not sure if a list item applies to your research, read the appropriate section before selecting a response.

Materials & experimental systems

n/a	Involved in the study	
☒	☐	Antibodies
☒	☐	Eukaryotic cell lines
☒	☐	Palaeontology and archaeology
☒	☐	Animals and other organisms
☐	☒	Human research participants
☐	☒	Clinical data
☒	☐	Dual use research of concern

Methods

n/a	Involved in the study	
☒	☐	ChIP-seq
☒	☐	Flow cytometry
☒	☐	MRI-based neuroimaging

Human research participants

Policy information about studies involving human research participants

Population characteristics	Table 2 shows characteristics of patients in training and validation datasets.
Recruitment	Retrospective collection of already available (and published) clinical trials.
Ethics oversight	The Institutional Review Board at the Montreal Neurological Institute (MNI), Quebec, Canada (Reference number: IRB00010120).

Note that full information on the approval of the study protocol must also be provided in the manuscript.

Clinical data

Policy information about clinical studies

All manuscripts should comply with the ICMJEguidelines for publication of clinical research and a completedCONSORT checklist must be included with all submissions.

Clinical trial registration

This study is not a prospective clinical trial but a retrospective analysis of 19 studies some of which are published clinical trials. The table 1 shows references to published papers of these studies. The clinical trials are registered in clinicaltrials.gov

The registration codes in clinicaltrials.gov for individual datasets mentioned in our manuscript are as follows

Siena Not registered.
Basel Not registered.
DEFINE- CONFIRM, ENDORSE NCT00420212
OPERA 1 NCT01247324
OPERA 2 NCT01412333
ASCEND NCT01416181
Lipoic acid NCT01188811
MS-STAT 1 NCT00647348
MAESTRO 3 NCT00468611
Lamotrigine NCT00257855
ARPEGGIO NCT02284568
INFORMS NCT00731692
PROMISE Not registered.
OLYMPUS NCT00087529
CLIMB Not registered.
ORATORIO NCT01194570
BRAVO NCT00605215
MS-SMART NCT01910259
MAESTRO1 and 2 NCT00869986

Study protocol	Not applicable for this study as a whole. However, individual studies have already published the protocols can be found above and also in publications shown in Table 1.
Data collection	Retrospective analysis of all major clinical trials in multiple sclerosis (MS), with a focus on progressive MS, over the past two decades (1996 to 2018). There are more than 770 centres whose data are part of our retrospective dataset from more than 100 countries. Data were aggregated at University College London and processed between 2017 to 2020.
Outcomes	We used a well-established disability measure widely used in clinical trials (24-week confirmed progression of Expanded Disability Status Scale or EDSS). The 24-week confirmed disability progression is a well-established outcome measure in clinical trials of multiple sclerosis.
